# Secretory Carcinoma of the Breast with Apocrine Differentiation—A Peculiar Entity

**DOI:** 10.3390/medicina60060924

**Published:** 2024-06-01

**Authors:** Anca Evsei, Adelina-Lucretia Birceanu-Corobea, Mihai Ghita, Narcis Copca

**Affiliations:** 1 Department of Pathology, Saint Mary Clinical Hospital, 011192 Bucharest, Romania; 2 Faculty of Medicine, Carol Davila University of Medicine and Pharmacy Bucharest, 050474 Bucharest, Romania; 3 Pathoteam Diagnostic, Pathology Laboratory, 051923 Bucharest, Romania; 4 Department of Surgery II, Saint Mary Clinical Hospital, 011192 Bucharest, Romania

**Keywords:** breast, secretory, apocrine differentiation

## Abstract

*Background and Objectives*: Secretory carcinoma of the breast is an uncommon histological subtype of breast cancer. There is little research on this entity and only a few larger studies, which lack consensus. We aim to report a particular apocrine differentiation in this subtype and ponder upon the clinical outcome of this case. *Case presentation*: We report the case of a 72-year-old female patient who presented to our hospital with a suspicious breast tumor. Core biopsy and mastectomy showed a low-grade breast carcinoma, a secretory subtype with apocrine differentiation. Immunohistochemistry confirmed both the secretory nature and the apocrine nature of the tumor cells. Surgical excision was considered curative and the patient is under long-term surveillance for any recurrences. *Conclusions*: There is very little research on the clinical behavior of secretory carcinomas with apocrine differentiation. The clinical outcome is unknown and, unfortunately, besides surgery, no other adjuvant treatments have shown efficacy. Further studies on long-term clinical progression are required for this rare entity.

## 1. Introduction

Secretory carcinoma of the breast is an unusual entity, with <0.05% of cases reported in the literature [1]. This histological subtype was primarily described in children [2], but recent studies have shown a prevalence in the fourth–seventh decades of life [1,3]. Most cases are identified in the upper-outer quadrant of the breast or near the nipple, but this subtype can be found in any part of the breast, including in ectopic breast tissue in the axilla [4]. These tumors are typically solitary, yellowish, well-circumscribed, and firm, and they are frequently mistaken for benign tumors (such as fibroadenoma) on ultrasound or mammography [5]. The microscopic features of this subtype are distinctive from no special-type breast carcinomas. The presence of secretory material, both intracellularly and extracellularly, is typical and a Nottingham grade 1 or 2 is frequent. The tumor cells are usually polygonal with eosinophilic and sometimes bubbly cytoplasm [6] and a low mitotic activity. Secretory ductal carcinoma in situ is often present [7].

Secretory carcinomas do not usually express ER, PR, and HER2, so they were initially classified as triple-negative carcinomas or basal-like carcinomas [8]. Nowadays, there are reported cases that express ER and PR, and most of them have a very good clinical prognosis compared to triple-negative or basal-like carcinomas [9]. Other immunohistochemical markers that are typically expressed are polyclonal CEA, S100, MUC4, and CK5/6 [3,5,7,10]. Molecular testing provides a clear diagnosis for secretory carcinomas. These cancers are characterized by the ETV6-NTRK3 gene fusion and are generally tested using RT-PCR or FISH techniques [8,9,11]. Immunohistochemical testing using pan-TRK monoclonal antibody shows promise, but further specificity is required to routinely use this technique for diagnosis [12].

Differential diagnosis may include breast carcinoma with apocrine differentiation, which is AR positive or acinic cell carcinoma [13]. Secretory carcinomas share an indolent clinical course with other low-grade breast carcinomas, with a 5-year 94% survival rate [14]. Treatment management usually includes lumpectomy or mastectomy with axillary lymph node excision. Chemotherapy or radiotherapy has not shown any noticeable benefit [15,16,17]. Novel molecule inhibitors that target NTRK3 and other NTRK family members have been developed and some have shown efficiency in breast secretory carcinomas [18,19]. Long-term surveillance is mandatory after surgical and oncological management, as this subtype is prone to late recurrences, especially in older patients [20].

This study aims to describe an unusual histopathological feature in these tumors. Secretory breast carcinoma with apocrine differentiation is scarcely documented in the literature, with only a handful of cases being reported [21,22]. Our review of case studies did not reveal any cases with apocrine features or AR (Androgen receptor) positivity (Table 1). It is unclear whether this histopathological peculiarity influences the clinical course of this subtype, but so far, most cases have shown a good clinical outcome.

## 2. Case Report

A 72-year-old female patient was admitted to our institution for a clinically palpable breast tumor located in the upper inner quadrant of the left breast (September 2022). Imaging studies (ultrasonography) revealed a BIRADS 4a tumor. Clinical examination showed a high body mass index and epigastric pain. The patient’s medical history consisted of liver cirrhosis with hepatitis virus C (2011), esophageal varices, portal hypertension, and arterial hypertension, all currently under treatment. The patient also suffered from COVID-19 in 2020 and 2021, and was vaccinated in 2021 following the approved protocols. Prior surgical procedures included an appendectomy, an excision of a left ovarian cyst, an extrauterine pregnancy, and a total hysterectomy with bilateral adnexectomy for a uterine leiomyoma.

**Figure 1:**
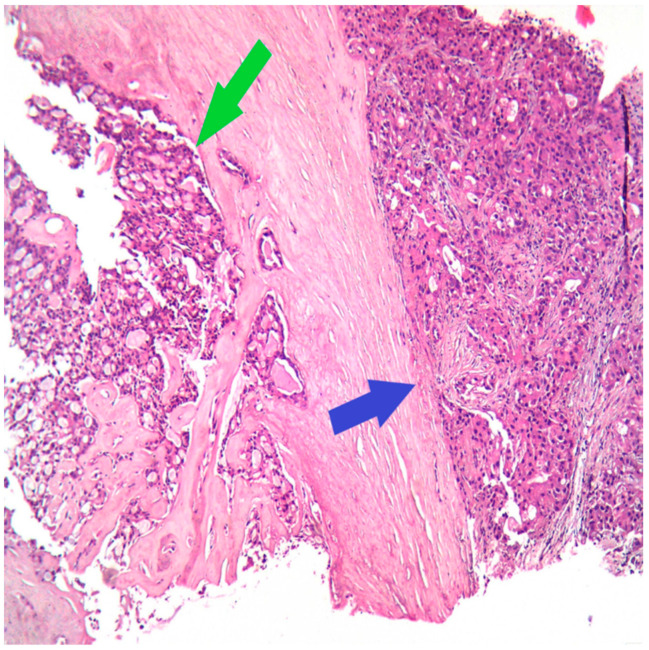
HE (Hematoxylin–Eosin stain), Ob 20×: Secretory carcinoma of the breast (green arrow) with apocrine differentiation (blue arrow) on the core biopsy.

The next step for this patient was the breast biopsy. Pathological examination showed an infiltrative tumor with polygonal tumor cells with eosinophilic or vacuolated cytoplasm, rare, atypical mitoses, and round nuclei; the growth pattern was mixed, microcystic and solid (Figure 1).

**Figure 2:**
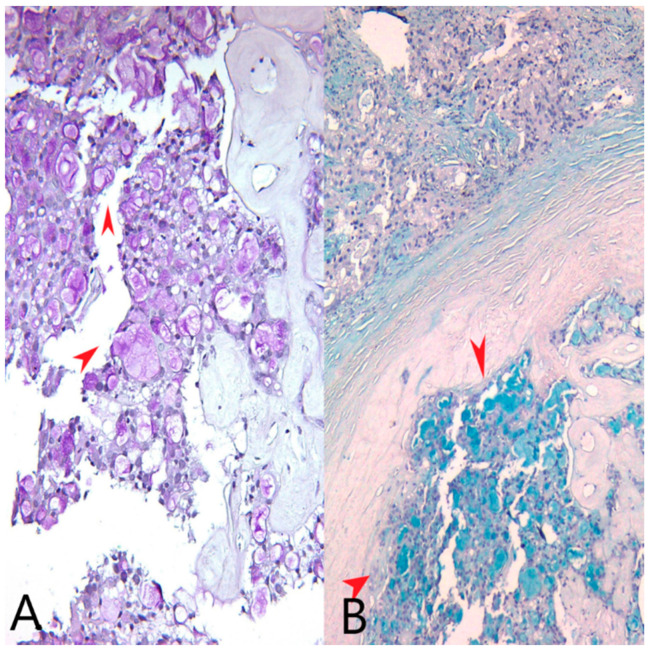
(**A**) PAS stain, Ob20×: Pink extracellular and intracellular secretions in tumor cells (red arrows). (**B**) Alcian blue stain, pH = 1.5, Ob20×: Bluish extracellular and intracellular secretions in tumor cells (red arrows).

**Figure 3:**
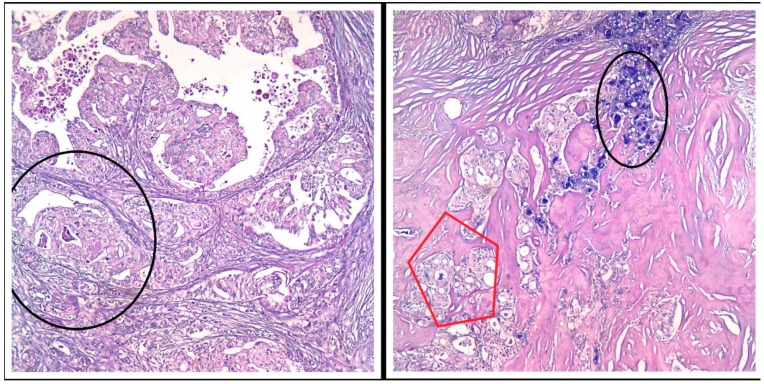
PAS/AB stain, Ob 20×: Both tumor components (the secretory and the apocrine component) express various amounts of pink (PAS stain) and bluish (AB pH = 1.5) intracellular secretions—black circles and red pentagon.

We identified extracellular and intracytoplasmic eosinophilic secretions that stained positively with PAS stain (Periodic acid–Schiff stain) and Alcian blue, pH = 1.5 stain (Figure 2). We also performed an AB/PAS stain (Alcian Blue pH = 1.5/Periodic acid–Schiff stain) on the mastectomy specimen (Figure 3).

Differential diagnosis came to mind between a secretory carcinoma, a carcinoma with apocrine differentiation, and an acinic cell carcinoma. We decided to perform an immunohistochemical study on the breast biopsy.

We chose the biopsy paraffin block for the immunohistochemical study. Sections were cut at 4 μm onto superfrost slides, preheated in a 60-degree oven for a minimum of 30 min. After labelling the slides and choosing the antibodies, we started with antigen retrieval; we used PT Link and envision Target retrieval solution high pH (50×) and low pH (50×) DAKO. We pressed RUN; this usually takes 30 min at a high pH to complete and 20 min at a low pH to complete. The detection kit used was DAKO-envision flex kit, high pH (link). The incubation time for the antibodies is 30 min using a manual approach and 20 min using an autostainer; after preparing the required reagents, we loaded the slide in the DAKO autostainer. We checked all the reagents and then we pressed START. After the program finished its cycle, we removed the slides from the racks and transferred them to the racks for the coverslipper. We placed the slides in water to wash for 5 min, then dehydrated them with 70% Alcohol, using two changes of Absolute Alcohol and two changes Xylene. We coverslipped the slides manually. The immunohistochemical panel included the following:

- ER, EP1 clone, Ready to Use (RTU), Dako
- PR, PgR636 clone, RTU, Dako
- AR, Ar441 clone, Concentrated, Dilution 1:50, Dako
- Ki67, MIB-1 clone, RTU, Dako
- Her2, c-erbB-2 oncoprotein, Concentrated, Dilution 1:200, Dako
- S-100, 4C4.9, Concentrated, Dilution 1:150, Zeta
- Gata-3, L50-823, Concentrated, Dilution 1:100, Zeta
- CEA, Polyclonal RPab, Concentrated, Dilution 1:250, Biosb
- CK 5/6, D5/16 B4 clone RTU, Dako.

ER, PR, AR, CK5/6, S100 and GATA3—a high pH was used in the PT Link; KI67, Her2, CEA Polyclonal—a low pH was used in the PT Link.

**Figure 4:**
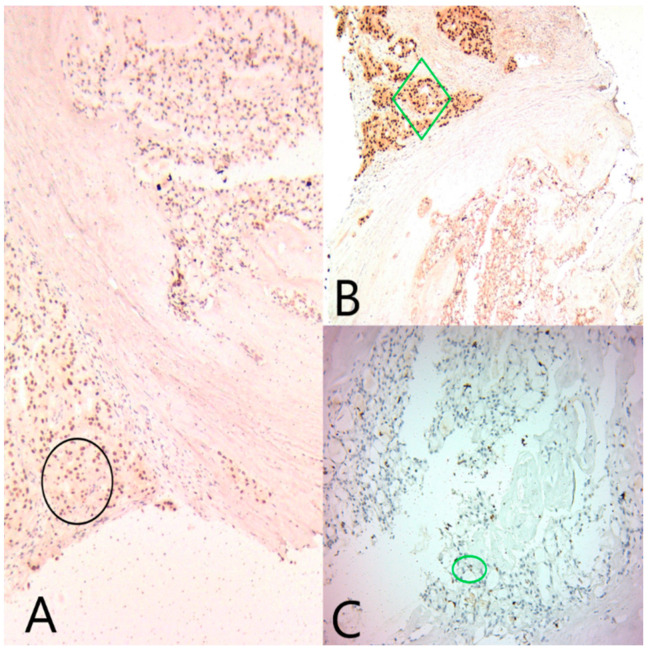
(**A**) Ob20x: GATA3 is diffusely positive in both tumor components (black circle). (**B**) Ob20×: AR is diffusely positive in the apocrine component (green diamond), and negative in the secretory component. (**C**) Ob20×: ER is focally positive in the secretory component (green circle).

GATA3 was diffusely positive in all tumor cells, confirming the mammary origin of the tumor. ER and PR were moderately positive in the microcystic component (Alred score = 4, H score = 4), and negative in the solid component. AR was negative in the microcystic component and diffusely positive in the solid component (Figure 4).

**Figure 5:**
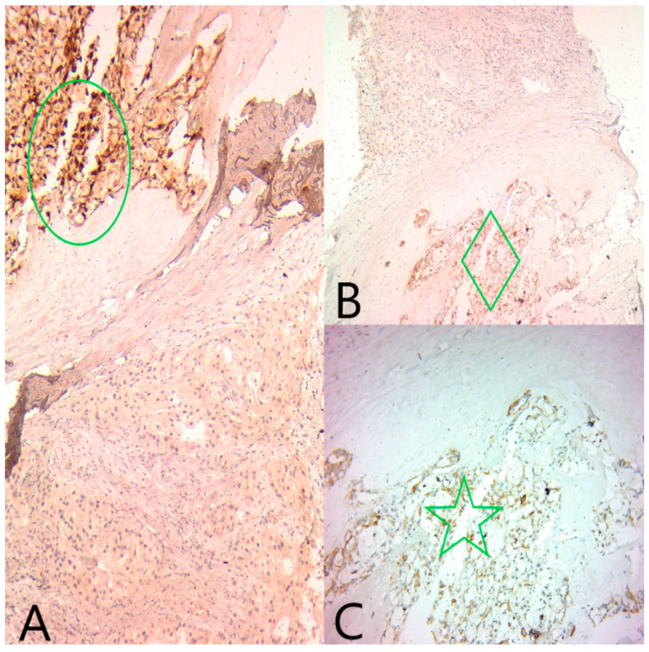
(**A**) Ob20×: S100 is diffusely positive in the secretory component (green circle) and negative in the apocrine component. (**B**) Ob20×: Polyclonal CEA is diffusely positive in the secretory component (green diamond) and negative in the apocrine component. (**C**) Ob20×: CK5/6 is diffusely positive in the secretory component (green star) and negative in the apocrine component.

CK5/6, CEA and S100 (Figure 5) were positive in the microcystic component and negative in the solid component. Ki67 was expressed in 14% of the nuclei in all tumor cells.

The final pathological and immunohistochemical diagnosis was secretory carcinoma of the breast with apocrine differentiation, well-differentiated (G1). To our knowledge, FISH testing showing the ETV6 rearrangement or identification of ETV6-NTRK3 by next-generation sequencing was not performed for this patient.

**Figure 6:**
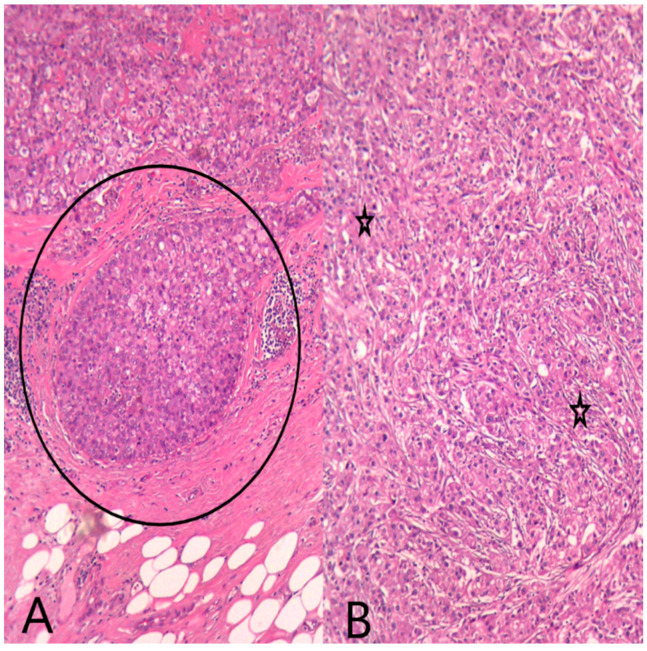
HE (Hematoxylin–Eosin stain) (**A**) Ob20×: Secretory ductal carcinoma in situ (DCIS) (black circle) on the mastectomy specimen. (**B**) Ob20×: Invasive breast carcinoma with apocrine morphology (black stars) on the mastectomy specimen.

The multidisciplinary team decided that the next step for this patient would be a radical mastectomy with regional lymph node excision. Gross examination showed a solitary, firm, circumscribed tumor with a yellowish tan cut surface, located in the inner quadrant of the breast. The tumor size was 1.8/1.2/0.8 cm. All resection margins were negative. Microscopic examination showed a tumor with similar characteristics to those on the breast biopsy. We also identified secretory ductal carcinoma in situ (DCIS), lympho-vascular invasion, perineural invasion, and 10% tumor-infiltrating lymphocytes (Figure 6). All axillary lymph nodes were negative.

The surgical treatment was considered curative and the patient was discharged. The patient was referred to an oncology specialist for further treatment. The oncologist decided to adopt a “wait-and-see” policy, as the patient was not eligible for adjuvant chemotherapy. Endocrine therapy was considered as a second line of treatment. Follow-up more than one year after the diagnosis shows that the patient is currently alive and under long-term surveillance.

## 3. Discussion

Secretory carcinoma of the breast was first described in patients younger than 20 years old, but nowadays this histological subtype has been reported in various decades of life (3–91 years), in both female and male patients [2]. Although this tumor may occur anywhere in the breast, reports show a predilection for the central quadrant in both young and male patients, as breast tissue is mostly localized in this region [11]. Our case presented with an inner-upper quadrant localization. This site is considered the second most common location, but for secretory carcinomas, it is fairly unusual [14,26]. After reviewing the literature, we found that the outer-upper quadrant of the breast is the most common location (Table 1).

Imaging studies such as ultrasonography or mammography are usually not very specific in pinpointing the type of lesion. Often enough, the tumor is suspected to be benign, such as in a fibroadenoma, a papilloma, a granular cell tumor, or even PASH [27]. Malignant differential diagnosis may include other types of invasive carcinomas or even phyllodes tumors. Most reports show that in sonographically, this tumor may present as a solid, hypoechoic lesion with a smooth lobulated border [23]; microcalcification has been rarely described. Our case was interpreted as a BIRADS 4 lesion, so the probability of a malignant diagnosis on histopathological examination was around 20–35%; therefore, the radiologist recommended a core biopsy of the tumor.

Gross examination usually finds a grayish-white or a tan-yellow tumor, with a firm consistency, lobulated, smooth borders, and sometimes with microcystic areas [24]. The microscopic examination identifies on core biopsy or mastectomy a tumor proliferation composed of large apocrine cells with eosinophilic cytoplasm, central nuclei with visible nucleoli, and medium secretory cells, with pale and amphophilic cytoplasm and abundant secretion. Most tumors have unequal percentages of these cells, but sometimes apocrine tumor cells may predominate and raise a differential diagnosis of carcinoma with apocrine features [21]. Our case presented with both cell populations, so our immunohistochemical panel includes markers for differential diagnosis. The secretory cells usually secrete an eosinophilic material that stains with PAS and Alcian blue special stains [2]. It is believed that this secretory material is composed of sulfated mucopolysaccharides and sialomucin [28], leading to a differential diagnosis with cystic hypersecretory lesions. In most cases, we find an intraductal component, which is often described on excision specimens and helps in confirming the secretory nature of this tumor [10,16,17].

Immunohistochemically, this subtype expresses polyclonal CEA, S100, and MUC 4, among other markers, often in a diffuse pattern [1]. These markers are useful for the diagnosis of histological subtypes, but more studies are needed to support this evidence. Most tumors were thought to be triple negative (negative ER, PR, and HER2) and therefore treated as such. Recent case reports however may show weak positivity for ER and PR, similar to our case [9,11], Positivity for CK5/6 reveals a basal-like phenotype, and our case was also positive [9]. AR is reported as being negative in secretory carcinomas [2] (Table 1). Most authors did not test for the androgen receptor, so there is not a clear picture for apocrine differentiation. One case [15] tested for this hormone receptor and it was negative. We tested this marker because of the apocrine features of the case, and it was positive. The literature search did not reveal case reports with diffuse AR positivity in secretory carcinomas with areas of apocrine differentiation. We believe that this differentiation is very peculiar and our concern was whether this tumor would have a better prognosis similar to a secretory carcinoma or a worse one similar to a carcinoma with apocrine differentiation [1].

ETV6-NTRK3 gene fusion is pathognomonic for this subtype of cancer. Most cases of secretory breast carcinomas with this type of NTRK fusion result from the translocation of DNA between ETV6 on the short arm of chromosome 12 (12P13.2) and NTRK3 on the long arm of chromosome 15 (15q25.3) to form a chromosome 15 derivative. The gene fusion encodes a chimeric protein containing the catalytic tyrosine kinase domain of NTRK3 (TRKC) linked to the sterile alfa motif dimerization domain of ETV6 [29].

Testing nowadays includes next-generation sequencing or FISH testing [1]. Immunohistochemistry for pan-TRK is not available for routine use and more reliable antibodies are necessary for secretory carcinomas. One study [30] showed the low specificity of detecting NTRK1-3 fusions using panTRK immunohistochemistry in breast carcinomas (82%) compared to other tumor types. The authors also compared immunohistochemistry to next-generation sequencing (NGS) in detecting NTRK1-3 fusions. They found an 87.9% sensitivity and an 81.1% specificity for pan-TRK immunohistochemistry, and an 81.1% sensitivity and a 99.86% specificity for the DNA-based cancer gene panel NGS. However, at the moment, there are many limitations regarding the testing of these fusions. Although NGS is probably the most specific, it is expensive and it may not identify all fusions present in the sample [31].

PanTRK immunohistochemistry is the most cost-efficient method and, if implemented, would offer a reliable biomarker for NTRK3 inhibitors. Some authors [32] found that a diffuse/focal strong nuclear positivity for the panTRK marker has a high sensitivity and specificity for breast secretory carcinomas. Consequently, a new line of treatment such as inhibitors that target NTRK3 would be considered for treating secretory carcinomas [19]. One recent study performed comprehensive genomic profiling on both secretory and non-secretory breast carcinomas. Interestingly enough, they confirmed that most secretory carcinoma showed fusion for ETV6-NTRK3 and that very few showed fusion for LMNA-NTRK1 fusion; on the other hand, they found NTRK fusions in non-secretory breast carcinomas, especially NTRK1. These data suggest that we may test for NTRK fusion on all breast carcinomas in the future, seeing as there are new inhibitors for NTRK available [33].

The prognosis for these tumors is usually favorable, especially in younger patients [1,28]. Even patients with axillary metastases have shown a good clinical course [9]. Most patients are treated with surgical mastectomy and some of them remain disease-free [2]. Our patient has not developed any recurrences more than a year after her surgery. Because this type of tumor can be more aggressive in older patients, the clinician may expect recurrences, so strict follow-up is mandatory. Adjuvant radiotherapy has been applied in some cases, and most studies have shown improved survival by combining surgery and radiotherapy [16]. Chemotherapy, on the other hand, is not commonly recommended, as this histological subtype is usually resistant to chemotherapy [25]. A few studies have mentioned the use of chemotherapy regimens in more aggressive tumors but with little or no success [34,35]. The most exciting line of treatment is represented by NTRK inhibitors. Larotrectinib and entrectinib have been used in solid tumors with NTRK fusions, but there are only a few studies that show the effect of this treatment on secretory breast carcinoma. The treatment results are often excellent [32]. However, we have to keep in mind the fact that currently, there are no large studies dealing with clinical results, treatment resistance or side effects in rare breast cancer subtypes.

## 4. Conclusions

Secretory breast carcinomas are unusual and rare tumors in the breast. Histopathological diagnosis and differential diagnosis are crucial, as this type has an indolent behavior and surgical excision is often sufficient. Although they were considered triple-negative carcinomas, it is important to keep in mind the fact that they usually behave as hormone-positive tumors. There is little to no research on AR positivity in secretory carcinomas with apocrine features. Therefore, the clinical outcome and prognosis are unknown, and convincing evidence will be needed in the future to support individualized clinical treatment.

## Figures and Tables

**Table 1:** Review of case studies.

Authors, Year	Number of Cases, Age, Sex	Breast Quadrant	Surgical Management	Gross Examination	ER, PR, HER2	CK5/6, s100, CEA	ETV6–NTR3 Translocation	Oncological Treatment	Recurrence, Metastasis
Shukla et al., 2020 [3].	1/60, F	Upper outer quadrant	FNA, radical mastectomy	Lobulated, well circumscribed, pushing margins	Triple negative	positive	present	None	None
Shin et al., 2001 [4]	1/46, F	Ectopic breast tissue in axilla	Incisional biopsy, 1 lymph node metastasis	Lobulated, well circumscribed, pushing margins	Triple negative	Not performed	Not performed	Adjuvant chemotherapy with Adriamycin, cyclophosphamide	None
Li D et al., 2012 [6]	15 cases (2 M, 13 F, median age 36	Upper outer quadrant	Lumpectomy in 5 cases Simple mastectomy and sentinel lymph node biopsy in one case Modified radical mastectomy in nine patients.	Grossly, two cases showed obscure boundaries and infiltrating margins, whereas others were well demarcated and non-encapsulated.	Triple negative	80% positivity	Not performed	Seven cases received postoperative chemotherapy, and one of them also had radiotherapy.	None
Lijuan et al, 2019 [5]	44 cases, F 7 cases < 30 years 37 cases over 30 years	Upper outer quadrant	Thirty-eight patients underwent modified radical mastectomy or radical mastectomy, and the remaining patients underwent conservative surgery. Lumpectomy was performed in two children (an 11-year-oldboy and a 4-year-old girl) and simple mastectomy with axillary lymph node dissection in three patients.	Painless and firm mass. The cut sections appeared greyish white to tan or yellow	47.7% (21/44) positivity for ER 52.3% (23/44) positivity for PR	positive	88.6% (39/44) positivity	40 patients underwent postoperative chemotherapy, and eleven had radiotherapy. Of 44 patients, 15 (34.1%) had positive axillary lymph nodes	Six SBC patients demonstrated evidence of recurrence and distant metastasis; five patients died from cancer
Yuan-Yuan Zhao, 2023 [10]	52 cases, 47 F 5 M, Mean age 46	Upper outer quadrant	Mostly radical mastectomy	Not evaluated	Triple-negative breast cancer (65.4%).	Not performed	16 of 18 cases (88.9%)	Not evaluated	None
Horowitz et al, 2012 [14]	83 cases, 81 F 2 M Median age 53	Upper outer quadrant	Lumpectomy 29.2% Mastectomy 70.8%	Not evaluated	Triple negative	Not performed	Not performed	35 patients (42.2%) received adjuvant radiation. Among the 39 patients treated initially with lumpectomy, 25 patients (64.1%) received radiation.	None
Tang et al., 2021 [15]	39, F	Upper outer quadrant	Modified radical mastectomy (left breast mastectomy and left axillary lymphadenectomy)	white grey multiple nodules and obvious accompanied by bleeding and necrosis.	Triple negative, AR negative	positive	positive	Neoadjuvant chemotherapy was then performed with 4 cycles of Epirubicin/Cyclophosphamide (EC) regimens and 2 cycles of Docetaxel/Carboplatin (DC) regimens	None
Gong et al., 2021 [17]	190 cases, Median age 56 years	Upper-outer quadrant (UOQ) irrespective of laterality	Breast conservative surgery Simple Mastectomy	slow-growing, painless, well-circumscribed, mobile mass	Positive staining of estrogen receptor (ER) and progesterone receptor (PR) was 58% and 40%	Not performed	Not performed	Yes 17 cases	None
Sharma et al., 2015 [22]	55, F	Upper quadrant	Lumpectomy Modified radical mastectomy with axillary dissection	Not evaluated	Triple negative	Not performed	Not performed	6 cycles of combination of Adriamycin, Cyclophosphamide, and Paclitaxel	None
Wu I-K et al, 2021 [23]	26, F	Lower inner quadrant	Surgical excision	Not evaluated	Triple negative	Positive	Not performed	None	Disease-free after 1 year
Aktepe et al, 2016 [24]	24, F	Not specified	segmental mastectomy with sentinel lymph node biopsy for right axilla.	well-circumscribed, greyish-white, rounded, and lobulated.	3% ER positivity PR, HER2 negative	Positive	Not performed	None	None
Lombardi et al., 2013 [25]	59, F		Surgical procedure of right breast quadrantectomy with homolateral axillary sentinel lymph node (SLN) biopsy	painless, with well-defined margins and increased in density	ER, HER2 negative PR 4% positivity	Not performed	Not performed	adjuvant radiotherapy	None

## Data Availability

The data that support the findings of this study are available from the corresponding author upon reasonable request.

## References

[1] Organisation Mondiale de la Santé (2019). Breast Tumours.

[2] Hoda S.A.F., Koerner F.C., Brogi E., Koerner F.C. (2021). Rosen’s Breast Pathology.

[3] Shukla A., Arshad F., Naseem I. (2020). Secretory carcinoma of breast: A diagnostic dilemma. Indian J. Pathol. Microbiol..

[4] Shin S.J., Sheikh F.S., Allenby P.A., Rosen P.P. (2001). Invasive Secretory (Juvenile) Carcinoma Arising in Ectopic Breast Tissue of the Axilla. Arch. Pathol. Lab. Med..

[5] Li L., Wu N., Li F., Li L., Wei L., Liu J. (2019). Clinicopathologic and molecular characteristics of 44 patients with pure secretory breast carcinoma. Cancer Biol. Med..

[6] Li D., Xiao X., Yang W., Shui R., Tu X., Lu H., Shi D. (2012). Secretory breast carcinoma: A clinicopathological and immunophenotypic study of 15 cases with a review of the literature. Mod. Pathol..

[7] Yang Y., Wang Z., Pan G., Li S., Wu Y., Liu L. (2019). Pure secretory carcinoma in situ: A case report and literature review. Diagn. Pathol..

[8] Laé M., Fréneaux P., Sastre-Garau X., Chouchane O., Sigal-Zafrani B., Vincent-Salomon A. (2009). Secretory breast carcinomas with ETV6-NTRK3 fusion gene belong to the basal-like carcinoma spectrum. Mod. Pathol..

[9] Del Castillo M., Chibon F., Arnould L., Croce S., Ribeiro A., Perot G., Hostein I., Geha S., Bozon C., Garnier A. (2015). Secretory Breast Carcinoma: A Histopathologic and Genomic Spectrum Characterized by a Joint Specific ETV6-NTRK3 Gene Fusion. Am. J. Surg. Pathol..

[10] Zhao Y.-Y., Ge H.-J., Yang W.-T., Shao Z.-M., Hao S. (2024). Secretory breast carcinoma: Clinicopathological features and prognosis of 52 patients. Breast Cancer Res. Treat..

[11] Krings G., Joseph N.M., Bean G.R., Solomon D., Onodera C., Talevich E., Yeh I., Grenert J.P., Hosfield E., Crawford E.D. (2017). Genomic profiling of breast secretory carcinomas reveals distinct genetics from other breast cancers and similarity to mammary analog secretory carcinomas. Mod. Pathol..

[12] Gatalica Z., Xiu J., Swensen J., Vranic S. (2019). Molecular characterization of cancers with NTRK gene fusions. Mod. Pathol..

[13] Reis-Filho J.S., Natrajan R., Vatcheva R., Lambros M.B.K., Marchio C., Mahler-Araújo B., Paish C., Hodi Z., Eusebi V., Ellis I.O. (2008). Is acinic cell carcinoma a variant of secretory carcinoma? A FISH study using ETV6 ‘split apart’ probes. Histopathology.

[14] Horowitz D.P., Sharma C.S., Connolly E., Gidea-Addeo D., Deutsch I. (2012). Secretory carcinoma of the breast: Results from the survival, epidemiology and end results database. Breast.

[15] Tang H., Zhong L., Jiang H., Zhang Y., Liang G., Chen G., Xie G.E. (2021). Secretory carcinoma of the breast with multiple distant metastases in the brain and unfavorable prognosis: A case report and literature review. Diagn. Pathol..

[16] Min N., Zhu J., Liu M., Li X. (2022). Advancement of secretory breast carcinoma: A narrative review. Ann. Transl. Med..

[17] Gong P., Xia C., Yang Y., Lei W., Yang W., Yu J., Ji Y., Ren L., Ye F. (2021). Clinicopathologic profiling and oncologic outcomes of secretory carcinoma of the breast. Sci. Rep..

[18] Shukla N., Roberts S.S., Baki M.O., Mushtaq Q., Goss P.E., Park B.H., Gundem G., Tian K., Geiger H., Redfield K. (2017). Successful Targeted Therapy of Refractory Pediatric ETV6-NTRK3 Fusion-Positive Secretory Breast Carcinoma. JCO Precis. Oncol..

[19] Lange A., Lo H.-W. (2018). Inhibiting TRK Proteins in Clinical Cancer Therapy. Cancers.

[20] Krausz T., Jenkins D., Grontoft O., Pollock D.J., Azzopardi J.G. (1989). Secretory carcinoma of the breast in adults: Emphasis on late recurrence and metastasis. Histopathology.

[21] Anderson P., Albarracin C.T., Resetkova E. (2006). A Large, Fungating Breast Mass. Arch. Pathol. Lab. Med..

[22] Sharma V., Anuragi G., Singh S., Patel P., Jindal A., Sharma R.G. (2015). Secretory Carcinoma of the Breast: Report of Two Cases and Review of the Literature. Case Rep. Oncol. Med..

[23] Wu I.-K., Lai Y.-C., Chiou H.-J., Hsu C.-Y. (2021). Secretory carcinoma of the breast: A case report and literature review. J. Med. Ultrasound.

[24] Aktepe F., Sarsenov D., Ozmen V. (2016). Secretory Carcinoma of the Breast. J. Breast Health.

[25] Lombardi A., Maggi S., Bersigotti L., Lazzarin G., Nuccetelli E., Amanti C. (2013). Secretory breast cancer. Case report. Il G. Chir.—J. Ital. Surg. Assoc..

[26] Castillo G.G.P., García R.C., Piña V.B., Andrade J.A.S., Jones J.E.M., Aziz A.M. (2019). Secretory breast carcinoma: A report of two cases. Rev. Senol. Patol. Mamar..

[27] Bhayana A., Misra R.N., Bajaj S.K., Bankhar H. (2018). Clinicoradiologicial aspects of secretory carcinoma breast: A rare pediatric breast malignancy. Indian J. Radiol. Imaging.

[28] Tavassoli F.A. (1999). Pathology of the Breast.

[29] Tognon C., Knezevich S.R., Huntsman D., Roskelley C.D., Melnyk N., Mathers J.A., Becker L., Carneiro F., MacPherson N., Horsman D. (2002). Expression of the ETV6-NTRK3 gene fusion as a primary event in human secretory breast carcinoma. Cancer Cell.

[30] Solomon J.P., Linkov I., Rosado A., Mullaney K., Rosen E.Y., Frosina D., Jungbluth A.A., Zehir A., Benayed R., Drilon A. (2020). NTRK fusion detection across multiple assays and 33,997 cases: Diagnostic implications and pitfalls. Mod. Pathol..

[31] Mortensen L., Ordulu Z., Dagogo-Jack I., Bossuyt V., Winters L., Taghian A., Smith B.L., Ellisen L.W., Kiedrowski L.A., Lennerz J.K. (2021). Locally Recurrent Secretory Carcinoma of the Breast with NTRK3 Gene Fusion. Oncologist.

[32] Medford A.J., Oshry L., Boyraz B., Kiedrowski L., Menshikova S., Butusova A., Dai C.S., Gogakos T., Keenan J.C., Occhiogrosso R.H. (2023). TRK inhibitor in a patient with metastatic triple negative breast cancer and NTRK fusions identified via cell-free DNA analysis. Ther. Adv. Med. Oncol..

[33] Wilson T., Sokol E.S., Ross J.S., Maund S.L. (2020). 131P NTRK1/2/3 fusions in secretory versus non-secretory breast cancers. Ann. Oncol..

[34] Benabu J.-C., Stoll F., Koch A., Molière S., Bellocq J.-P., Mathelin C. (2018). De-escalating systemic therapy in triple negative breast cancer: The example of secretory carcinoma. J. Gynecol. Obstet. Hum. Reprod..

[35] Balkenhol M.C.A., Vreuls W., Wauters C.A.P., Mol S.J.J., van der Laak J.A.W.M., Bult P. (2020). Histological subtypes in triple negative breast cancer are associated with specific information on survival. Ann. Diagn. Pathol..

